# Recent Studies on the Insulin-Secreting Porosome Complex Provide Potential Therapeutic Applications in the Treatment of Diabetes

**DOI:** 10.15190/d.2015.43

**Published:** 2015-12-04

**Authors:** Lloyd L. Anderson

**Affiliations:** Department of Animal Science, College of Agriculture and Life Sciences and Department of Biomedical Sciences, College of Veterinary Medicine, Iowa State University, Ames, IA 50011-3150 USA

**Keywords:** Porosome, Insulin Secretion, Islet Transplant, Diabetes

In type 1 diabetes, insulin-secreting β-cell replacement by islet transplant from cadaveric organ donor pancreas, carried out for decades, is potentially a curative therapy. Unfortunately, the function of the transplanted β-cell in the transplanted islets progressively declines, due in part to impairment in their secretory function. Studies report^1-3^ that, as early as one week following islet transplant, insulin secretion is significantly lower than in freshly isolated islets, and greatly reduced at 40 weeks. Furthermore, the current islet isolation protocols are unable to recover all islets from the entire pancreas, further limiting the number of transplanted islets, resulting in an insufficient β-cell mass for the recipient.

**Figure 1:**
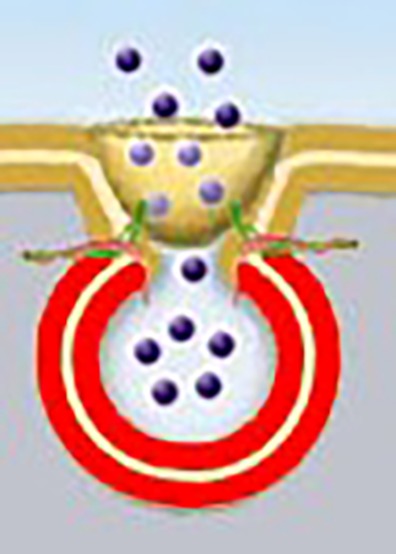
Figure 1. Schematic drawing of a cup-shaped porosome complex at the cell plasma membrane with a fused secretory vesicle (red) at its base facing the cytosolic compartment of the cell. Note the arrangement of t-/v-SNAREs in a rosette or ring complex to establish the fusion pore or continuity at the porosome base.

Nearly 20 years ago, permanent cup-shaped lipoprotein structures at the cell plasma membrane, called “porosomes”, were discovered (see schematic representation of the porosome in**Figure 1**), where secretory vesicles transiently dock and fuse to expel intravesicular contents from the cell. Porosomes are present in all secretory cells, from the digestive enzyme-secreting pancreatic acinar cells, to the hormone-releasing growth hormone and insulin-secreting cells, mast cells, chromaffin cells, hair cells of the inner ear, and in neurons secreting neurotransmitters^4,5^. Porosomes have been immunoisolated from a number of cells including the exocrine pancreas and neurons, biochemically characterized, and functionally reconstituted into artificial lipid membrane^5^. A large body of evidence has accumulated on the role of porosome-associated proteins on cell secretion and secretory defects, including neurotransmission and neurological disorders^6^. In a recent study, the porosome complex in mouse insulinoma Min6 cells was isolated, its proteome determined^7^, and the isolated porosome was functionally reconstituted into live Min6 cells^8^.

Mass spectrometry on isolated Min6 porosome demonstrates the presence of 30 core proteins including SNAREs, the heat shock protein Hsp90, the calcium-transporting ATPase type 2C, and the potassium channel subfamily K member 2. In a recently published study^8^, isolated Min6 porosomes reconstituted into live Min6 cells demonstrate augmented levels of porosome proteins and a consequent increase in the potency and efficacy of glucose-stimulated insulin secretion. Elevated glucose-stimulated insulin secretion 48 h post reconstitution, reflects the remarkable stability and viability of reconstituted Min6 porosomes, documenting for the first time the functional reconstitution of native porosomes into live cells and their potential utility in therapy. However, further studies in an animal model will be required to determine the viability and function of transplanted islets reconstituted with the isolated insulin-secreting porosome complex. It is important to note that the porosome discovery has far reaching implications in health and medicine. For example, mass spectrometry on isolated porosomes from human lung epithelial cells demonstrates interaction between the cystic fibrosis trans-membrane conductance regulator (CFTR) and the porosome complex in human airways epithelia, shedding light on the possible regulatory role of CFTR on the quality of mucus secretion via the porosome complex^9^. Reconstitution of functional porosome complex isolated from cadaveric human lung epithelia could potentially ameliorate secretory defects in patients with the CFTR disease. Similarly, post-traumatic stress disorder or PTSD in part attributed to loud explosions, or continuous white noise, has been demonstrated to result in memory loss and anxiety, among others health detriments. Ultrastructure studies on the neuronal porosome complex in rats subjected to continuous white noise report alterations in the neuronal porosome morphology, hence its chemistry^10^. Therefore, the important discovery of the porosome complex has come full circle: from elucidation of its structure and dynamics in all secretory cells examined, to its isolation, determination of its chemistry, its structural and functional reconstitution into artificial lipid membranes, to finally its functional reconstitution into live secretory cells, and the identification of its important participation in health and disease, resulting in a paradigm-shift in our understanding of the secretory process. Great future applications await the pioneering discovery of the porosome complex, analogous to the proteasome, ribosome, the lysosome, or the nuclear pore complex.

- **The discovery of the porosome complex has come full circle: from elucidation of its structure and dynamics in secretory cells examined, to its structural and functional reconstitution into artificial lipid membranes and secretory cells, and the identification of its important participation in health and disease**.
